# The Consumption of Beer

**Published:** 1917-07-14

**Authors:** 


The Hospital

The Workers' Newspaper of Administrative Medicine and Institutional Life, Administration, National Insurance and Health.

No. 1623, Vol. LXII. SATURDAY, JULY 14, 1917. PRICE ONE PENNY

### THE CONSUMPTION OF BEER.

We are pleased to be able to record that the Government has authorised an increased output of beer for the quarter ending September 30 next. The Hospital has for many years (*vide* its report on whiskey, April 7, 1906; light wines, June 15, 1907; and beer, April 24, 1909) taken a special interest in the subject of alcoholic beverages, and has endeavoured, and to some extent succeeded, in advocating true temperance, building its arguments not upon the ephemeral catchwords of faddists, but upon the durable basis of scientific consideration. We, therefore, make no apology for putting before our readers some of the reasons why the action of the Government meets with our approval. In doing this we shall initially state the case against the increased production of beer in the present crisis, which was capable of far more forcible exposition than its adherents presented in the House of Commons. These gentlemen one and all bombarded the Government from under the shelter of the catch-word "drink." Even the heavy artillery of infant mortality was used to its full, and the public was informed that the brewing of an extra million barrels of beer in the ensuing quarter must be regarded as deliberately aiding one of the principal causes of infant mortality. It might be argued with equal readiness that the man who took an extra bath a day from the Thames should be convicted of tampering with the water supply of London.

The main argument against the increased production of beer, and it is worthy of the most serious consideration, is that in the present shortage of cereals and sugar it is wasteful, in so far as the nutritive value of the manufactured article is less than the sum of the nutritive values of its constituents. As a fact, the brewers are not to have any more sugar, and they are not actually to have any more malt, but they are to be allowed to use more of the existing stock of malt in the present quarter. The wastage argument is one which is applicable to all culinary processes. Most foodstuffs, at any rate from the chemical standpoint, waste on being cooked. Especially is this true if we take into consideration the fuel required for the cooking. Brewing is essentially cooking; the preparation of a special "broth" from malt and other constituents. Perhaps one of the most extraordinary instances of waste is bread. Civilised man demands his main stock of cereal food in the shape of leavened bread. The chemical loss of nutritive elements in the leavening of bread is very considerable. Apart from the breeding of yeast, which has to be as carefully done as the breeding of horses and is relatively as extravagant, and about which we may incidentally remark there are at present very considerable difficulties, some of which at least are due to the diminished beer production; dough loses from 2.1 to 4.2 per cent. of its nutritive material by being leavened by yeast. Practically the whole of this is represented by the production of alcohol. It may cause joy to the teetotaller, but will certainly cause woe to the economist to know that Graham estimated that 300,000 gallons of alcohol were lost yearly in London in the leavening of bread by yeast. Put in another way, we shall be very much under the estimate if we assume that enough bread is lost in this country daily to feed 400,000 individuals on a ration of ½ lb. per diem. Nevertheless the mass of the population refuse to eat unleavened bread or bread leavened by other methods than yeast.

It cannot be too definitely insisted upon that the nutritive value of any food or drink is not what it contains, but what the body, with a reasonable output of energy, can get out of it. Further, complicated nervous processes dependent upon the smell, taste, colour, and general appearance of food alter with the amount of energy-giving substances we can extract from a given food, and also the amount of energy which we have to expend to extract them. The whole art of gastronomics finds its justification in these principles. We habitually take with meals, to help us to enjoy and digest them, substances whose intrinsic nutritive value may be low. They act like oil in machinery; they are not fuel, but it would be difficult to utilise fuel without them. A mass of the population of extreme importance find it very unpleasant and extremely difficult to do without beverages of low alcoholic content—*e.g.*, beer. The machinery of their daily life runs easier, and their forced labour is less irksome with them than without them. Brain workers and manual workers alike, from the fulness of their experience, have been forced to this considered conclusion. In so far as we are dependent upon their work, their food, even if some of it is the product of somewhat expensive cookery, should be a recognised charge upon the food assets of the nation. Beer is a beverage proved by generations of experience to have special properties. It contains nutritive material, it assuages thirst, it is free from disease germs, and in those to whom it appeals it adds a relish to and thus stimulates the digestion of their meals. The quantity of beer brewed in 1914 was 36,000,000 standard barrels. According to the Home Secretary, the amount to be allowed during this, the thirstiest, quarter of the present year, is to be under 4,000,000 standard barrels, so that it cannot be said that the beer drinker has not endeavoured to deny himself during the present crisis. The diminution is actually greater than appears from these figures because the quality of the beer is not the same.

We feel sure that all who can view this matter stripped of the distortion with which the faddists attempted to clothe it will come to the conclusion, that the Government have acted in the best interests of the State in apportioning a quantity of food material slightly in excess of their original estimate for the nation's workers. They have acted with judgment and circumspection, and we venture to hope that they will exercise these same attributes when the sweet and chocolate question is further considered. That the working man should have no beer, but that the young lady, imbued with the laudable desire to know the inwardness of things, should console herself *ad lib.*, while following the mental turmoils of Brieux or Ibsen, with the sweets of Fuller seems to the ordinary observer as reprehensible as it is anomalous.

